# Preparation of Hot-Melt-Extruded Solid Dispersion Based on Pre-Formulation Strategies and Its Enhanced Therapeutic Efficacy

**DOI:** 10.3390/pharmaceutics15122704

**Published:** 2023-11-30

**Authors:** Seon-Kwang Lee, Eun-Sol Ha, Heejun Park, Kyu-Tae Kang, Ji-Su Jeong, Jeong-Soo Kim, In-hwan Baek, Min-Soo Kim

**Affiliations:** 1 College of Pharmacy, Pusan National University, 63 Busandaehak-ro, Geumjeong-gu, Busan 46241, Republic of Korea; lsk7079@pusan.ac.kr (S.-K.L.); edel@pusan.ac.kr (E.-S.H.); sui15@pusan.ac.kr (J.-S.J.); 2 College of Pharmacy, Duksung Women’s University, 33, Samyangro 144-gil, Dobong-gu, Seoul 01369, Republic of Korea; heejunpark@duksung.ac.kr (H.P.); ktkang@duksung.ac.kr (K.-T.K.); 3 Dong-A ST Co., Ltd., Giheung-gu, Yongin 17073, Republic of Korea; 4 College of Pharmacy, Kyungsung University, 309, Suyeong-ro, Nam-gu, Busan 48434, Republic of Korea; baek@ks.ac.kr

**Keywords:** hot-melt extrusion, solid dispersion, miscibility, molecular interaction, bisacodyl, in vivo efficacy

## Abstract

In this study, an amorphous solid dispersion containing the poorly water-soluble drug, bisacodyl, was prepared by hot-melt extrusion to enhance its therapeutic efficacy. First, the miscibility and interaction between the drug and polymer were investigated as pre-formulation strategies using various analytical approaches to obtain information for selecting a suitable polymer. Based on the calculation of the Hansen solubility parameter and the identification of the single glass transition temperature ($T_{g}$), the miscibility between bisacodyl and all the investigated polymers was confirmed. Additionally, the drug–polymer molecular interaction was identified based on the comprehensive results of dynamic vapor sorption (DVS), Fourier transform infrared spectroscopy (FT-IR), Raman spectroscopy, and a comparison of the predicted and experimental values of $T_{g}$. In particular, the hydroxypropyl methylcellulose (HPMC)-based solid dispersions, which exhibited large deviation between the calculated and experimental values of $T_{g}$ and superior physical stability after DVS experiments, were selected as the most appropriate solubilized bisacodyl formulations due to the excellent inhibitory effects on precipitation based on the results of the non-sink dissolution test. Furthermore, it was shown that the enteric-coated tablets containing HPMC–bisacodyl at a 1:4 ratio (*w*/*w*) had significantly improved in vivo therapeutic laxative efficacy compared to preparations containing un-solubilized raw bisacodyl in constipation-induced rabbits. Therefore, it was concluded that the pre-formulation strategy, using several analyses and approaches, was successfully applied in this study to investigate the miscibility and interaction of drug–polymer systems, hence resulting in the manufacture of favorable solid dispersions with favorable in vitro and in vivo performances using hot-melt extrusion processes.

## 1. Introduction

Most developed pharmaceutical drugs and approximately 90% of drug candidates are poorly water-soluble, resulting in low dissolution rates and oral bioavailability. Therefore, the solubilization of poorly water-soluble drugs is important in the development of drug products. Various solubilization techniques exist, including amorphous solid dispersion, co-amorphous systems, eutectic mixtures, nanosuspension, inclusion complexes (e.g., cyclodextrins), and various lipid-based formulations, including micelle, emulsion, and self-emulsifying drug delivery systems [1,2,3,4,5,6]. Among the various solubilization techniques, a solid dispersion system is defined as a drug dispersed in a solid matrix, which is generally a polymer. Solid dispersions can be manufactured with a relatively simple system consisting of a polymer and a drug to improve the oral bioavailability of poorly water-soluble drugs by maintaining the amorphous form of the drug within the solid dispersion [1,7]. Among the various processing techniques for manufacturing amorphous solid dispersions, such as solvent evaporation, the kneading method, anti-solvent precipitation, the co-precipitation method, spray-drying, lyophilization/freeze-drying, and supercritical fluid technologies, the hot-melt extrusion process is a solvent-free operation that avoids stability risks from residual amounts of solvent and offers many advantages, such as ease of scale-up, low cost, and the potential for continuous manufacturing. In addition, the hot-melt extrusion process facilitates the preparation of solid dispersions, because fewer processing steps are required, which classically include mixing, melting, solidification during extrusion, and downstream processes. Nevertheless, it has several disadvantages, such as thermal degradation of heat-sensitive materials, high start-up costs, and the requirement of specialized knowledge [8]. The preparation of amorphous solid dispersions processed by the hot-melt extrusion process is based on dispersing the drug into a molten polymer matrix under controlled conditions, such as the feeder speed, processing temperature, and screw speed.

For the successful development of drug products, especially an amorphous solid dispersion processed by the hot-melt extrusion, proper selection of the polymer in the early development stage of the amorphous solid dispersion, which is called the pre-formulation stage, is very important [9]. The optimal polymer for the hot-melt extrusion process should dissolve the drug substance in its matrix to form a stable amorphous solid dispersion without thermal degradation and be easily extrudable at the defined manufacturing processing conditions [10]. In addition, the detailed considerations that may be helpful in selecting a polymer are as follows: (1) the degradation temperature of the polymer must be at least 50 °C above its glass transition temperature ($T_{g}$); (2) the processing temperature during hot-melt extrusion might be kept above the $T_{g}$ of the polymer; and (3) the interaction and miscibility with the drug should exist to stabilize the amorphous state and enhance solubilization capacity [9,11]. Assessing the miscibility and molecular interaction of drug and polymer systems is crucial for selecting the optimal polymer because it is directly related to the solubility enhancement and stability of the amorphous solid dispersion. Various theoretical models and thermodynamic evaluations have been performed to select suitable polymers for solid dispersions processed by hot-melt extrusion [12,13].

In this study, bisacodyl was selected as a model drug with poor water-solubility (below 3.0 μg/mL in water at 37 °C, log *p* value of 3.45). The poor aqueous solubility of bisacodyl and the small amount of fluid present in the large intestine might reduce its therapeutic effect [14,15]. Therefore, for the desired laxative effect of bisacodyl, its solubilization is required. As a pre-formulation strategy for the preparation of an amorphous solid dispersion for the solubilization of bisacodyl, the miscibility and molecular interactions between commonly used polymers for the hot-melt extrusion process and bisacodyl were evaluated using differential scanning calorimetry (DSC), powder X-ray diffraction (PXRD), dynamic vapor sorption (DVS), Fourier transform infrared spectroscopy (FT-IR), and Raman spectroscopy. In addition, several theoretical models, including the Hansen solubility parameter, Flory–Huggins interaction parameter, and Gordon–Taylor equation, were applied for the prediction and fundamental understanding of the amorphous solid dispersion formation. In addition, the in vitro non-sink dissolution test was conducted to investigate the effect of miscibility and molecular interactions between the drug and polymer on the solubilization capacity. Furthermore, an in vitro dissolution test and in vivo therapeutic efficacy evaluation in constipation-induced rabbits were conducted for enteric-coated tablets containing bisacodyl amorphous solid dispersions.

## 2. Materials and Methods

### 2.1. Materials

Bisacodyl (0.999 in mass fraction purity) was kindly provided by Dong-A ST Co., Ltd. (Seoul, Republic of Korea). Hydroxypropyl cellulose (HPC, SSL grade) was obtained from Nippon Soda Co., Ltd. (Tokyo, Japan). Hydroxypropyl methylcellulose (HPMC, Pharmacoat^®^ 603), hydroxypropyl methylcellulose acetate succinate (HPMCAS, AQOAT^®^ MG), and hydroxypropyl methylcellulose phthalate (HPMCP, HP-50) were acquired from Shin-Etsu Chemical Co., Ltd. (Tokyo, Japan). Polyvinylpyrrolidone K12 (PVP K12, Kollidon^®^ K12), polyvinylpyrrolidone vinyl acetate copolymer (PVP VA64, Kollidon^®^ VA64), and polyvinyl caprolactam–polyvinyl acetate–polyethylene glycol graft copolymer (Soluplus^®^) were supplied by BASF Co., Ltd. (Ludwigshafen, Germany). Ultrapure water was obtained using the Milli-Q purification system (Millipore, Molsheim, France). Other chemicals and solvents used in this study were of high-performance liquid chromatography (HPLC) or reagent grade.

### 2.2. Differential Scanning Calorimetry (DSC)

Differential scanning calorimetry (DSC) was performed using a Discovery DSC 25 equipped with a refrigerated cooling system 90 (TA Instruments, Inc., New Castle, DE, USA) to characterize the thermal properties of bisacodyl, the polymers, and the physical mixtures. Physical mixtures between bisacodyl and different polymers were prepared by mixing in a plastic bag for 15 min. The DSC instrument was calibrated for temperature and enthalpy using an indium standard prior to the measurements. The samples were weighed to approximately 5 mg using an XS205 analytical balance (Mettler Toledo, Greifensee, Switzerland) and crimped in an aluminum pan. The samples were then heated from −10 to 170 °C at a scanning rate of 10 °C/min under a nitrogen atmosphere. In addition, modulated DSC (MDSC) was used to determine the $T_{g}$. The samples were equilibrated at −10 °C for 5 min and then heated from −10 to 170 °C at a scanning rate of 5 °C/min with modulation of ± 0.80 every minute, then cooled to −10 °C at a rate of 10 °C/min, followed by a second heating at a rate of 5 °C/min.

### 2.3. Dynamic Vapor Sorption (DVS)

Dynamic vapor sorption (DVS) analysis was performed using an SPSx-1μ Advanced system (ProUmid GmbH & Co. KG, Ulm, Germany) to investigate the interaction between bisacodyl and the polymers. Approximately 15 mg of the samples was loaded into a sample holder and then dried to a constant mass (relative humidity (RH) < 1%) at 25 °C for 3 h to remove residual water. Subsequently, samples were exposed to 10%, 20%, 30%, 40%, 50%, 60%, 70%, 80%, and 90% RH at 25 °C; then, the desorption cycle was conducted using the same profile. The time between the weighting cycles was set to 15 min. If the weighted mass change was less than 0.01% within 40 min, the next step was performed before the maximum time (50 h). The samples from the DVS experiments were further analyzed using PXRD to determine the stability of the amorphous solid dispersion.

### 2.4. Powder X-ray Diffraction (PXRD)

PXRD patterns of raw bisacodyl and bisacodyl containing solid dispersions were obtained using an Xpert 3 (Malvern Panalytical, Almelo, The Netherlands) with CuKα radiation (λ = 1.5406 Å) to assess the crystallinity of the solid dispersion prepared by the hot-melt extrusion process. Samples were scanned from 5° to 60° at a scanning rate of 3°/min. The tube voltage and current were set at 40 kV and 30 mV, respectively.

### 2.5. Fourier-Transform Infrared Spectroscopy (FT-IR) and Raman Spectroscopy

Fourier-transform infrared spectroscopy (FT-IR) analysis was conducted on a Nicolet iS50 spectrometer (Thermo Scientific, Madison, WI, USA) in the range of 4000–400 cm^−1^ with a resolution of 4 cm^−1^ to determine the intermolecular interactions between bisacodyl and the polymers. The samples were premixed with dry potassium bromide (KBr) using a mortar and pestle, followed by compression to prepare KBr disks. Raman spectra were collected using a Raman-HR-TEC spectrometer (StellarNet Inc., Tampa, FL, USA) with a diode laser for excitation at 785 nm and resolution of 4 cm^−1^. Experiments were conducted over the range between 200 and 2750 cm^−1^ based on the backscattering method.

### 2.6. In Vitro Non-Sink Dissolution Test

To evaluate the solubility capacity and supersaturation performance of bisacodyl containing a solid dispersion, the non-sink dissolution test was conducted using 200 mL dissolution flasks, mini paddles, and a VK 7000 dissolution tester (Agilent Technologies, Santa Clara, CA, USA) with a pH 7.2 buffer of 100 mL at a setting temperature and paddle speed of 37 °C and 150 rpm, respectively. The volume of pH 7.2 buffer was determined by considering the volume of the large intestinal fluid [14]. Bisacodyl-containing solid dispersions, equivalent to 5 mg of bisacodyl, were placed in a dissolution vessel. At predetermined time points, samples (1.5 mL) were withdrawn and then quickly filtered through a 0.45 µm filter fitted with a regenerated cellulose membrane (Agilent Technologies, Santa Clara, CA, USA), followed by dilution with methanol. The concentration of bisacodyl was determined using an HPLC system (Shimadzu, Tokyo, Japan) equipped with the solvent delivery module, UV/Vis detector, autosampler, and CAPCELL PAK C8 UG120 column (4.6 mm × 150 mm, 5 μm; Osaka Soda Co., Ltd., Osaka, Japan) [15]. The isocratic mobile phase was a mixture of 0.05 M potassium dihydrogen phosphate and acetonitrile at a ratio of 45:55 (*v*/*v*), with a flow rate of 1.0 mL/min and an injection volume of 20 μL. The detection wavelength and column temperature were set at 214 nm and 30 °C, respectively. Linearity was verified from 0.1 to 100 μg/mL, with an excellent determination coefficient (R^2^), corresponding to 0.9999. The dissolution experiments for each tablet were triplicated (*n* = 3).

### 2.7. Preparation of Bisacodyl Containing Solid Dispersion Using the Hot-Melt Extrusion Process

Bisacodyl solid dispersions were manufactured using a twin-screw HAAKE MiniLab II extruder (Thermo Scientific, Waltham, MA, USA) equipped with a co-rotating twin-screw with a diameter of 16 mm. Before processing, bisacodyl and different polymers were mixed in a plastic bag for 15 min. Powder blends of bisacodyl and the polymers were fed manually into a hot-melt extruder. The barrel temperature and screw speed were set at 140 °C and 100 rpm, respectively. Extrusion was performed without a die. The obtained extrudates were cooled at room temperature and milled into a fine powder using an A11 analytical mill (IKA, Staufen, Germany).

### 2.8. Preparation of Enteric-Coated Tablet Containing Bisacodyl Amorphous Solid Dispersion

The enteric-coated tablet containing the selected bisacodyl amorphous solid dispersion was prepared as reported earlier [14]. Table 1 represents the composition of the enteric-coated tablet formulation. Raw bisacodyl or hot-melt extruded solid dispersions (Bisacodyl:HPMC = 1:4 *w*/*w*) were blended with inactive ingredients using a V-mixer (Koreamedi Co. Ltd., Daegu, Republic of Korea) at 20 rpm for 10 min. The lubricated powder mixture with magnesium stearate was compressed into round convex tablets using a single-punch tablet press (Erweka GmbH, Frankfurt, Germany) equipped with a round-shape concave-type punch with a 6 mm diameter under a compression force of 12 ± 1 kN. For the preparation of the enteric-coating solution, the coating polymers, Eudragit L100 and S100 (Evonik Industries, Essen, Germany), were dissolved in the mixture of acetone and isopropanol (4:6 *w*/*w*) with TEC (Sigma Aldrich Co., St. Louis, MO, USA) and talc (Hwawon Pharm. Co., Ltd., Seoul, Republic of Korea) using a homogenizer (T-18 Basic ULTRA-TURRAX^®^, IKA^®^-WERKE GMBH & Co. KG, Staufen, Germany) at 4000 rpm. The total solute content in the final coating solution was 13.5%. The plain tablets were introduced in a pan coater (HCT-30 Hi coater, FREUND Industrial Co. Ltd., Tokyo, Japan); then, the prepared coating solution was sprayed onto the plain tablets through a nozzle with diameter of 1.2 mm (inlet air temperature: 50 °C; pan rotating rate: 45 rpm; spray rate: 5 mL/min). The hardness of the prepared enteric-coated tablets, determined using the crushing strength measurement method (Erweka, Heusenstamm, Germany), was 38 ± 2 kp.

### 2.9. In Vitro Dissolution Test of Enteric-Coated Tablet

The USP paddle method (VK 7000 dissolution testing station) was used for the dissolution testing of enteric-coated tablets (37 °C ± 0.1 °C, 100 rpm). The dissolution test was performed in a continuous dissolution medium. Firstly, the dissolution test was started in a buffer system at pH 1.2 (750 mL); then, the acidity of the dissolution medium was continuously changed to 6.4 after 2 h, 7.2 after an additional 1 h, and 6.7 after an additional 2 h, by addition of 0.5 M sodium phosphate solution (58 mL), 0.5 M sodium phosphate solution (15 mL), and 0.5 M hydrogen chloride solution (17 mL), respectively [14]. The applied pHs at 1.2, 6.4, 7.2, and 6.7 corresponded to the typical physiological pH conditions in the stomach, upper small intestine, lower small intestine, and colon, respectively. In addition, the experimental times at each pH condition for the dissolution test were set based on generally accepted gastrointestinal transit times. The number of dissolution experiments for each tablet was six. Then, 5 mL aliquots for each sample were collected at pre-determined time points, and an equal volume of the test medium was replaced. Sample solutions filtered through a syringe filter (0.45 μm, GF/C, Whatman, Maidstone, UK) were appropriately diluted with organic solvent (methanol) and then injected into a high-performance liquid chromatography (HPLC) system (LC-20AT, Shimadzu, Tokyo, Japan) equipped with a UV detector (SPD 20A, Shimadzu, Tokyo, Japan) for the determination of the drug concentration. A reverse phase C18 column (Zorbax Eclipse XDB^®^-C18, 150 × 4.6 mm i.d., 5 µm, Agilent Technologies, Santa Clara, CA, USA) was used for the chromatographic separation of bisacodyl. UV detection was set at 268 nm.

### 2.10. In Vivo Efficacy in Constipation-Induced Rabbits

The animal study protocol used for in vivo pharmacological assessment was in compliance with the institutional guidelines for the care and use of laboratory animals and was approved by the ethics committee of Kyungsung University (No. 18-023A, Approval Date: 14 October 2018). New Zealand white rabbits (3.0–3.5 kg, 8–10 weeks old) were purchased from Hyochang Science (Daegu, Republic of Korea). After a 7-day acclimatization with food and water ad libitum, loperamide hydrochloride dispersed in deionized water was administered orally to rabbits once a day for 3 days for constipation induction [14]. Rabbits whose stool weight for a day decreased by less than 60% compared to before treatment with loperamide were confirmed as constipated animals and were included in the evaluation of the in vivo laxative efficacy. Three groups (*n* = 8 per group), including enteric-coated tablets containing a drug-free vehicle (Vehicle-administered group), raw bisacodyl (F1-administered group), or solubilized bisacodyl (F2-administered group), were evaluated for in vivo therapeutic efficacy in constipation-induced rabbits at a single dose of 2.5 mg bisacodyl with 4 mL water. In addition, a group consisting of normal rabbits administered only water was also evaluated for comparison (Normal group). The fecal sample collection was performed for each rabbit at 6 h, 12 h, and 24 h after single-dose administration, and the stool weight at each time point was measured using an electric balance. In addition, the water content of the total fecal matter collected for a day was determined as the difference in the stool weights before and after oven drying for 12 h at 60 °C.

## 3. Results and Discussion

### 3.1. Pre-Formulation Strategies for Evaluation of Drug–Polymer Miscibility and Interaction

#### 3.1.1. Calculation of Hansen Solubility Parameter

Commonly used polymers in hot-melt extrusion for preparing solid dispersions, including $T_{g}$ and degradation temperatures, are presented in Table S1. Based on the values of glass transition and degradation temperatures, in this study, seven polymers (HPC, HPMC, HPMCAS, HPMCP, PVP K12, PVP VA64, and Soluplus^®^) were selected to evaluate the miscibility and interaction with bisacodyl, having a melting peak temperature of 135 °C, which was nearly identical to previously reported values [4,15]. To evaluate the miscibility and molecular interaction of the drug–polymer system, the Hansen solubility parameter was applied [10,16]. The Hansen solubility parameters of bisacodyl and the polymers were calculated using the Van Krevelen–Hoftyzer group contribution method [17,18].

Generally, if the difference in the Hansen solubility parameter between the drug and the polymer (Δδ) is less than 7.0 MPa^1/2^, it is considered good miscibility. However, if the difference in the solubility parameter is above 10 MPa^1/2^, the drug–polymer system is expected to be immiscible [10,19,20]. The solubility parameters estimated using the group contribution method for the drug and the polymers are listed in Table 2 and Table S1. The differences in the Hansen solubility parameters between bisacodyl and the polymers (HPC, HPMC, HPMCAS, HPMCP, PVP K12, PVP VA64, and Soluplus^®^) were 3.8 MPa^1/2^, 2.5 MPa^1/2^, 2.9 MPa^1/2^, 3.8 MPa^1/2^, 6.8 MPa^1/2^, 5.1 MPa^1/2^, and 2.8 MPa^1/2^, respectively. These results show that Δδ values were less than 7.0 MPa^1/2^ in the bisacodyl and seven polymer systems (HPC, HPMC, HPMCAS, HPMCP, PVP K12, PVP VA64, and Soluplus^®^), indicating good miscibility between bisacodyl and the polymers.

In addition, the Flory–Huggins interaction parameter between bisacodyl and the polymers was calculated using the Hansen solubility parameter. Based on Flory–Huggins theory, a numerical value close to zero of the interaction parameter leads to a small enthalpy and free energy of mixing, which indicates favorable mixing and intermolecular interaction within a drug–polymer system [10]. The interaction parameter (χ) can be expressed as follows:

(1) $$\chi =\frac{V_{site}}{RT}{\left(\delta_{drug}-\delta_{polymer}\right)}^{2}$$

where $\delta_{drug}$ and $\delta_{polymer}$ are the Hansen solubility parameters of the drug and polymer, respectively. $V_{site}$, *R*, and *T* denote the hypothetical lattice volume, gas constant, and absolute temperature, respectively [21].

The Flory–Huggins interaction parameters of bisacodyl and the polymers (HPC, HPMC, HPMCAS, HPMCP, PVP K12, PVP VA64, and Soluplus^®^) were found to be 1.29, 0.56, 0.75, 1.29, 4.13, 2.32, and 0.70, respectively. Therefore, the value of the interaction parameter was the lowest for HPMC, followed by Soluplus^®^ and HPMCAS.

#### 3.1.2. Comparison of Theoretical and Experimental Glass Transition Temperatures

**Figure 1:**
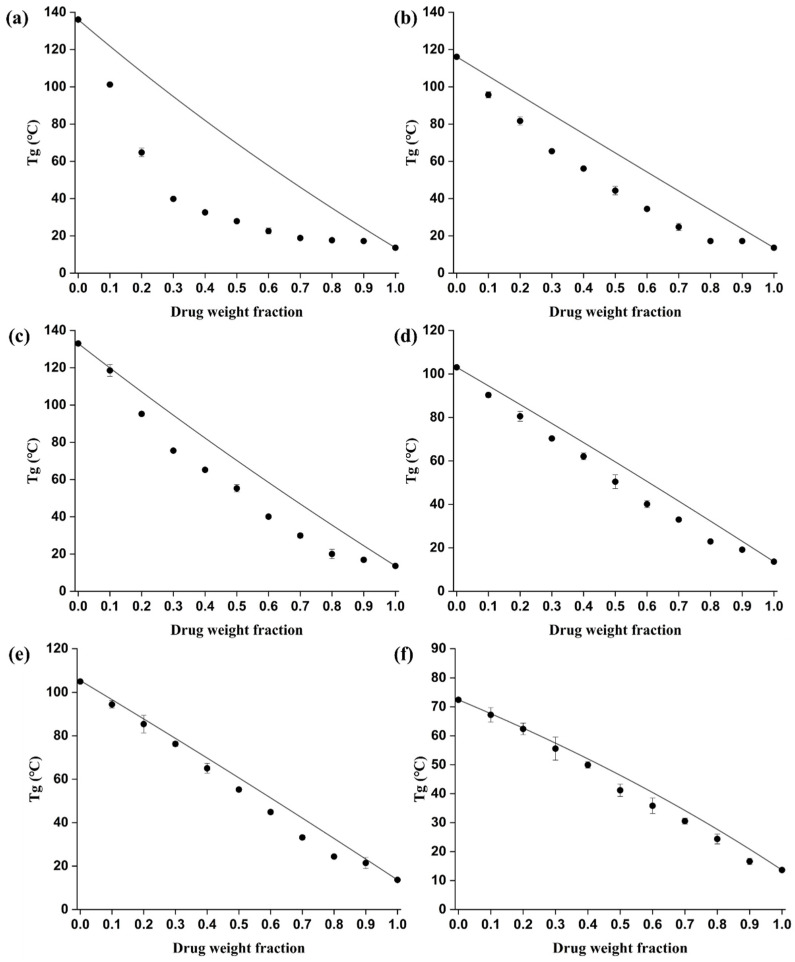
Comparison between the experimental (symbols) and predicted values (line) of the glass transition temperature ($T_{g}$) from the Gordon–Taylor equation: (**a**) bisacodyl–HPMC; (**b**) bisacodyl–HPMCAS; (**c**) bisacodyl–HPMCP; (**d**) bisacodyl–PVP K12; (**e**) bisacodyl–PVP VA64; (**f**) bisacodyl–Soluplus^®^.

Based on the Gordon–Taylor relationship, the $T_{g}$ of bisacodyl and the polymer mixture was determined using MDSC analysis to assess the miscibility and interaction between bisacodyl and the polymers. The Gordon–Taylor equation is as follows:

(2) $$T_{g}=\frac{w_{1}T_{g1}+Kw_{2}T_{g2}}{w_{1}+Kw_{2}}$$

where $T_{g1}$ and $T_{g2}$ denote the glass transition temperatures of bisacodyl and the six polymers (HPMC, HPCAS, HPMCP, PVP K12, PVP VA64, and Soluplus^®^), respectively. $w_{1}$ and $w_{2}$ are the weight fractions of bisacodyl and the six polymers in the physical mixtures, respectively. In the above equation, the K value denotes the ratio of the differences in the expansion coefficient (Δα) of bisacodyl and the polymers at the glass transition temperature. However, if using the weight fraction instead of the volume fraction and assuming that $\Delta \alpha T_{g}$ is constant, the K value is defined as follows [22]:

(3) $$K=\frac{\rho_{1}T_{g1}}{\rho_{2}T_{g2}}$$

where, $\rho_{1}$ and $\rho_{2}$ denote the true density of bisacodyl and the six different polymers, respectively. The predicted and experimental $T_{g}$ values of the mixture of bisacodyl and the six selected polymers are presented in Figure 1, except for HPC. The amorphous bisacodyl was produced by the hot-melt extruder of the crystalline form of bisacodyl. As shown in Figure S1, amorphous bisacodyl was determined by DSC analysis, which showed that the $T_{g}$ of bisacodyl was 13.6 °C and demonstrated an exothermic crystallization peak. In addition, the $T_{g}$ of the polymers HPMC, HPCAS, HPMCP, PVP K12, PVP VA64, and Soluplus^®^ were observed at 136.1 °C, 116.1 °C, 133.0 °C, 103.1 °C, 105.4 °C, and 72.4 °C, which were similar to the reported values in the literature [20,23,24,25,26]. Unfortunately, in this study, the $T_{g}$ of HPC could not be determined, which is a well-known problem of HPC [27,28]. This may be attributed to the complex morphological structure of HPC, which makes it difficult to identify its $T_{g}$ [29].

In general, a single $T_{g}$ is observed when the drug–polymer system is miscible; however, two separate glass transitions corresponding to each component are observed when the drug and polymer system is immiscible. From the results of the second heat ramp, a single $T_{g}$ was observed for all the physical mixtures of bisacodyl and the polymers, which increased as the weight ratio of the polymer in the physical mixtures increased. The appearance of a single $T_{g}$ between the glass transition temperature of bisacodyl and that of the polymer indicates good miscibility of all bisacodyl–polymer systems. In addition, the experimental $T_{g}$ values were found to be lower than those calculated using the Gordon–Taylor equation for all the physical mixtures. The maximum differences between theoretical $T_{g}$ and experimental $T_{g}$ of the physical mixtures of bisacodyl and the polymers HPMC, HPCAS, HPMCP, PVP K12, PVP VA64, and Soluplus^®^ were found to be 54.9 °C, 20.2 °C, 19.1 °C, 10.4 °C, 8.9 °C, and 5.2 °C, respectively. In particular, a large negative deviation was determined between the predicted and experimental values of $T_{g}$ in the bisacodyl–HPMC system compared with that of the other polymers. This is a dominant phenomenon in mixtures of low-molecular-weight organics and polymers and can be explicated in terms of the thermodynamics of mixing [30,31]. The interaction between amorphous drugs and polymers is related to the stabilization and maintenance of the amorphous state of the drug from crystallization. In addition, the physical stability of the amorphous state in the hot-melt-extruded solid dispersion prepared with the polymer may be explained by the steric hindrance effect owing to the structure of the polymer and the reduced diffusion of drug molecules. The reduced diffusion of drug molecules in hot-melt-extruded solid dispersions would inhibit the nucleation and growth of crystals owing to the anti-plasticization effect of the polymer, with relatively high viscosity and strong interactions between the drug and polymer, including hydrogen bonding. Thermodynamically, the interaction between the drug and polymer is related to the enthalpy of mixing. Stronger interactions between the drug and polymer could decrease the enthalpy of mixing, indicating favorable mixing and increasing the $T_{g}$ of the mixture. However, the mixing process of the drug and the polymer is affected not only by the enthalpy of mixing but also by the entropy of mixing related to the mixing volume [30]. When preparing a solid dispersion, a drug with a relatively small volume can easily penetrate the strands of the polymer and increase the distance between the polymer chains, thereby excessively increasing the mixing volume and entropy of mixing. In this case, the miscibility between the drug and polymer may be influenced by the steric hindrance effect (entropic) of the polymer as kinetic barrier that effectively inhibits nucleation and crystal growth as well as molecular interaction (enthalpic), including hydrogen bonding interaction between the drug and polymer [30,32]. Therefore, even if miscibility and interaction are ensured, the experimental $T_{g}$ may negatively deviate from the theoretical value obtained from the Gordon–Taylor equation.

### 3.2. Characterization of the Solid Dispersion Prepared by Hot-Melt Extrusion

#### 3.2.1. PXRD Analysis

Based on the above experiments in the pre-formulation strategies, bisacodyl solid dispersions prepared using seven polymers (HPC, HPMC, HPMCAS, HPMCP, PVP K12, PVP VA64, and Soluplus^®^) at a 1:2 drug:polymer ratio (*w*/*w*) were characterized using PXRD analysis. As the process conditions for preparing bisacodyl solid dispersion, the process temperature was set to 140 °C considering the melting temperature of bisacodyl, and the screw speed was set to 100 rpm in consideration of the equipment used [33]. The PXRD patterns of the raw bisacodyl and bisacodyl-containing solid dispersions are shown in Figure S2. In the PXRD diffractograms, several typical crystalline characteristic diffraction peaks were detected in the spectrum of raw bisacodyl, whereas typical halo patterns, indicating the amorphous phase, were observed in all the solid dispersions processed by hot-melt extrusion [34,35]. Therefore, from the results of the PXRD analysis, it was concluded that bisacodyl in solid dispersions at a 1:2 drug:polymer ratio (*w*/*w*) manufactured by the hot-melt extrusion process was dispersed in the used polymer matrix as an amorphous phase, as predicted in the pre-formulation study.

#### 3.2.2. DVS Analysis

DVS analysis can provide gravimetric information for samples at a well-controlled temperature and relative humidity. Various studies for amorphous materials, including identification of the content of the amorphous state in crystalline material, evaluation of the stability of amorphous material, and the molecular interaction between drug substances and polymers in solid dispersion, have been conducted based on the principle of DVS analysis [36]. Among the above purposes, to investigate the interaction between bisacodyl and the seven selected polymers (HPC, HPMC, HPMCAS, HPMCP, PVP K12, PVP VA64, and Soluplus^®^), water sorption of the amorphous form of bisacodyl, the polymers, their mixtures, and the solid dispersions were carried out using DVS analysis. In general, DVS was conducted to determine the affinity between water and the material. A typical water sorption isotherm, which reflects the interaction between water and the material, shows the mass change of the mixture caused by water uptake at a specific temperature and pressure [36,37,38]. The moisture sorption isotherms of non-interacting mixtures can be easily predicted using the following equation:

(4) $$W_{mix}=\frac{\left(W_{drug}m_{drug}+W_{polymer}m_{polymer}\right)}{\left(m_{drug}+m_{polymer}\right)}$$

where $m_{drug}$ and $m_{polymer}$ denote the mass of bisacodyl and the seven different polymers in the mixture, respectively. $W_{drug}$ and $W_{polymer}$ are the water uptake of bisacodyl and the seven polymers in each pure component, respectively. However, if molecular interactions are present between the bisacodyl and polymer, a deviation from the predicted water sorption isotherm might be observed. In addition, the magnitude of the deviation is affected by the strength of the molecular interaction between the drug and polymer system [38].

**Figure 2:**
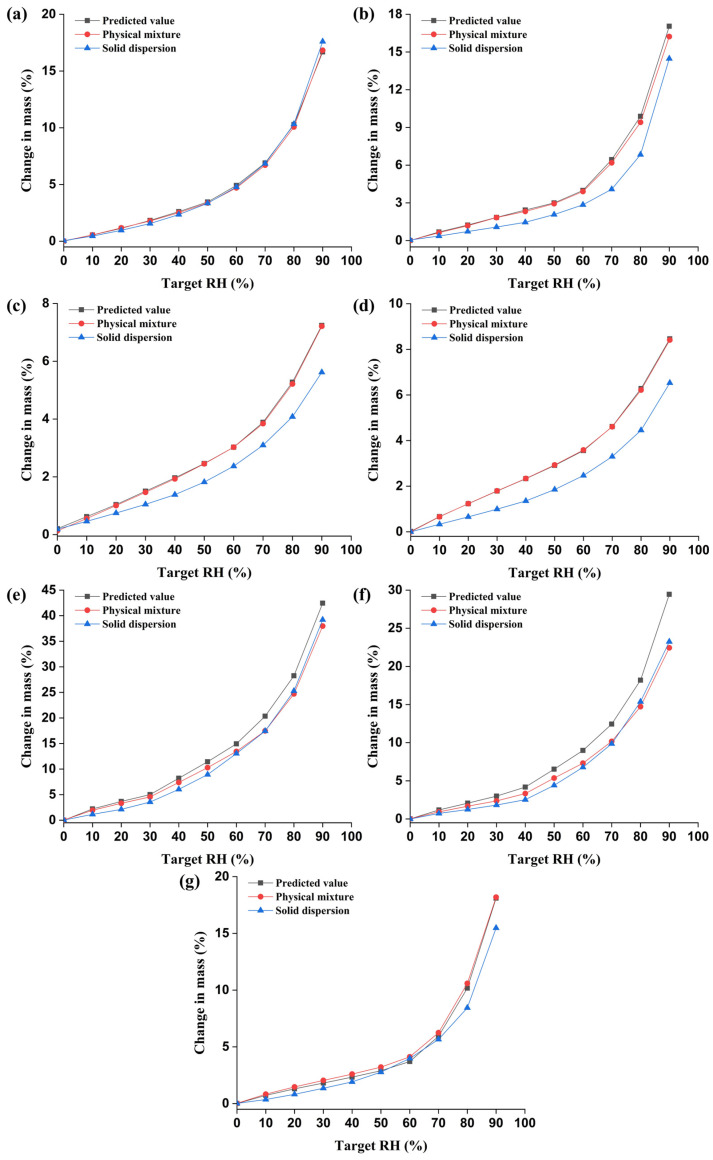
Predicted and experimental water sorption isotherms of amorphous solid dispersion prepared by hot-melt extrusion: bisacodyl:HPC (**a**), bisacodyl:HPMC (**b**), bisacodyl:HPMCAS (**c**), bisacodyl:HPMCP (**d**), bisacodyl:PVP K12 (**e**), bisacodyl:PVP VA64 (**f**), and bisacodyl:Soluplus^®^ (**g**) (weight ratio of drug:polymer = 1:2).

The water sorption isotherms of the amorphous bisacodyl, polymers, physical mixtures (amorphous bisacodyl:polymer ratio of 1:2 *w*/*w*), and solid dispersions at 25 °C are illustrated in Figure 2 and Figure S2. As shown in Figure S3, the changes in the masses of HPC, HPMC, HPMCAS, and HPMCP were 24.60%, 25.14%, 10.43%, and 12.26% at 90% RH, respectively. The weight gains of PVP K12, PVP VA64, and Soluplus^®^ at 90% RH were 63.24%, 43.74%, and 26.73%, respectively, which are nearly identical to the reported values [39]. For the amorphous form of pure bisacodyl, a weight gain of 0.85% was observed at 90% RH. As shown in Figure 2, by comparing the predicted isotherms with the experimental results of the pure components, it was observed that the water uptake decreased in all bisacodyl-containing solid dispersions, except for HPC. These results may have been caused by a decrease in the number of polar functional groups that could interact with water because of interactions such as the hydrogen bonding interaction between the drug and polymer [37,38]. The isotherms of the solid dispersions, such as bisacodyl–PVP K12 and bisacodyl–PVP VA64, prepared by the hot-melt extrusion process were nearly identical to those of their physical mixtures. These results may be attributed to the highly hygroscopic polymers (PVP K12 and PVP VA64), which intensively absorb water at high relative humidity and transform into a gel, which can easily interact with the drug [38].

**Figure 3:**
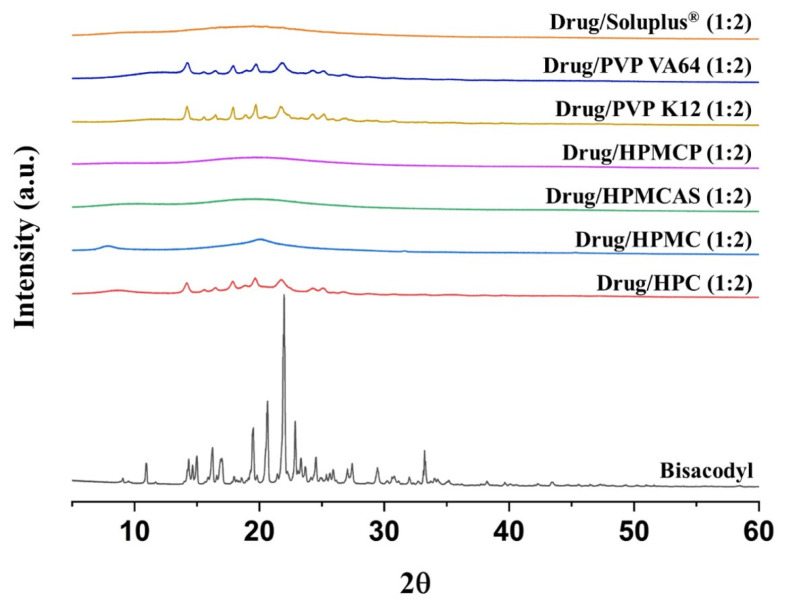
PXRD patterns of bisacodyl and DVS-treated solid dispersion prepared by hot-melt extrusion.

Furthermore, to evaluate the stability of bisacodyl-containing solid dispersions, samples from the DVS experiments were analyzed using PXRD [12,40,41]. Moisture sorption by DVS experiments would negatively influence the stability of the amorphous state in hot-melt-extruded solid dispersions by increasing the crystallization rate. This phenomenon may be caused by decreasing the glass transition temperature by increasing the uptake of moisture, which acts as a plasticizer. As the glass transition temperature decreases with water uptake, the diffusion and crystallization of drug molecules can be promoted by increased molecular mobility of the polymer [42,43]. As shown in Figure 3, the hot-melt-extruded solid dispersions prepared with HPC, PVP K12, and PVP VA64 showed characteristic peaks corresponding to crystalline bisacodyl, indicating that transformation from an amorphous phase to a crystalline form occurred after the DVS experiments. Unfortunately, the DVS experiments did not show an abrupt change in the slope of the isotherm as the amorphous drug recrystallized. This result may be attributed to the very low hygroscopicity of amorphous bisacodyl and the difficulty in observing the recrystallization of less than 0.5% amorphous content in DVS experiments [44]. In particular, these phase transitions in the bisacodyl-containing solid dispersions with PVP K12 and PVP VA64 may have been caused by the relatively high hygroscopicity of these polymers. However, the DVS-treated bisacodyl solid dispersions prepared with HPMCAS and HPMCP, which have low hygroscopicity, were nearly identical to those of the samples before the DVS experiments, which showed a halo pattern. In the HPMC-based solid dispersion and Soluplus^®^-based solid dispersion, which showed a similar profile to the water sorption isotherms of HPC, typical halo patterns were observed after DVS experiments, indicating no transformation from an amorphous state to a crystalline form. Therefore, based on these results, bisacodyl solid dispersions with HPMC, HPMCAS, HPMCP, and Soluplus^®^ were relatively stable compared to the solid dispersions prepared with HPC, PVP K12, and PVP VA64.

#### 3.2.3. FT-IR and Raman Analyses

**Figure 4:**
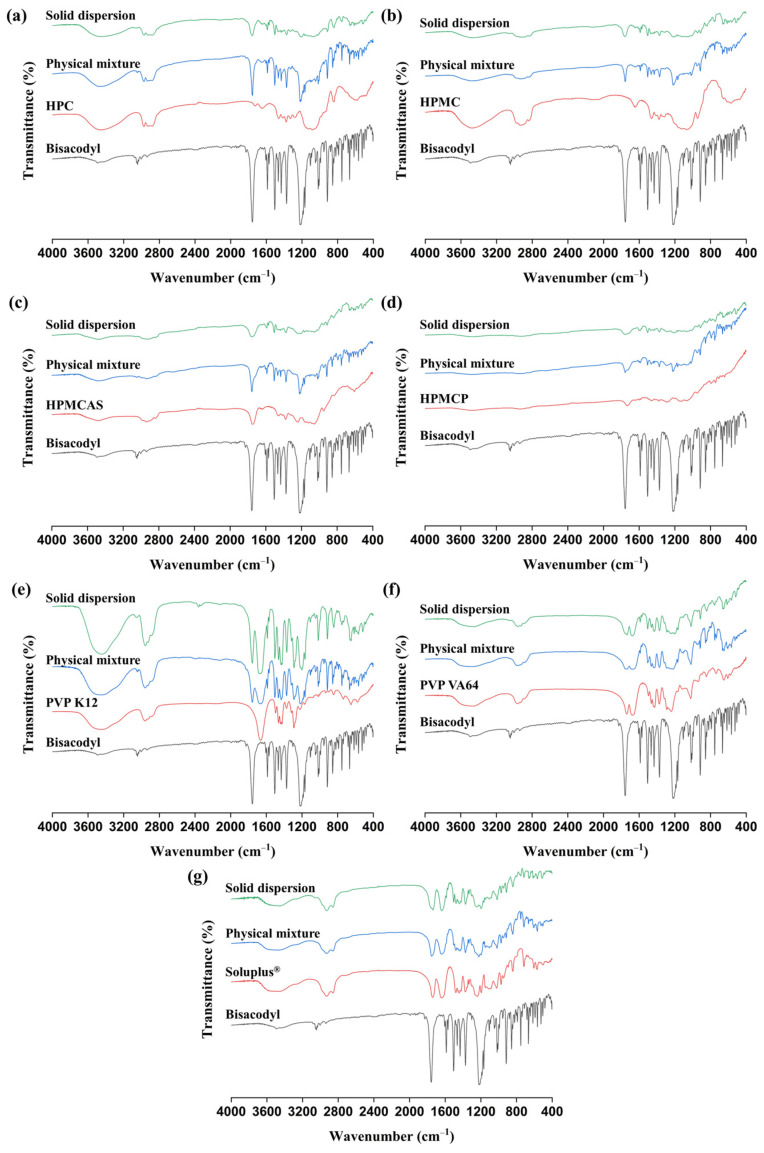
FT-IR spectra of pure bisacodyl, pure polymers, physical mixtures (drug:polymer = 1:2, *w*/*w*), and solid dispersions (drug:polymer = 1:2, *w*/*w*): (**a**) bisacodyl–HPC; (**b**) bisacodyl–HPMC; (**c**) bisacodyl–HPMCAS; (**d**) bisacodyl–HPMCP; (**e**) bisacodyl–PVP K12; (**f**) bisacodyl–PVP VA64; (**g**) bisacodyl–Soluplus^®^.

FT-IR analysis was conducted to determine the molecular interaction between bisacodyl and the polymer in the solid dispersion. As shown in Figure 4 and Table 3, the characteristic peak of pure bisacodyl is observed at 1757 cm^−1^, indicating the presence of a C=O group. The sharp peaks at 1506 cm^−1^, 1465 cm^−1^, and 1431 cm^−1^ were assigned to the vibration of the aromatic groups, whereas the characteristic peaks at 1218 cm^−1^ and 1208 cm^−1^ were assigned to the stretching vibrations of the C−O group [4,45]. In particular, the vibration peak of the C=O group at 1757 cm^−1^ weakened and broadened in the bisacodyl solid dispersions prepared with HPC, HPMC, HPMCAS, and HPMCP, indicating that the C=O groups of the drug were restricted by the polymers. In addition, the absence of aromatic group peaks (1465 cm^−1^ and 1431 cm^−1^) and C−O group peaks (1218 cm^−1^ and 1208 cm^−1^) was observed in the solid dispersions with cellulose derivatives, such as HPC, HPMC, HPMCAS, and HPMCP [4]. In contrast to the solid dispersion samples, a sharp peak of bisacodyl at 1757, 1465, and 1431 cm^−1^ was observed in the IR spectra of all the physical mixtures of the bisacodyl–polymer systems. This remarkable change in IR peak for amorphous solid dispersions can mean that the vibrational characteristics of the structure have changed due to interaction with the polymer, such as the hydrogen bond between the hydroxyl group of cellulose and the oxygen in the bisacodyl structure. In the PVP VA64–based and Soluplus^®^-based solid dispersions, peaks at 1218 cm^−1^ and 1208 cm^−1^ were absent, but the vibration peaks of aromatic groups at around 1450 cm^−1^ remained. This may mean a weaker interaction compared to the interaction between cellulose derivatives and bisacodyl as mentioned above, although it is generally very difficult to quantitatively assess the degree of interaction due to the broad peaks. It is supposed that this interaction might be the weakest in the PVP K12–based solid dispersion, because remarkable changes, such as the absence of a characteristic IR peak, were not observed in its FT-IR spectrum.

**Figure 5:**
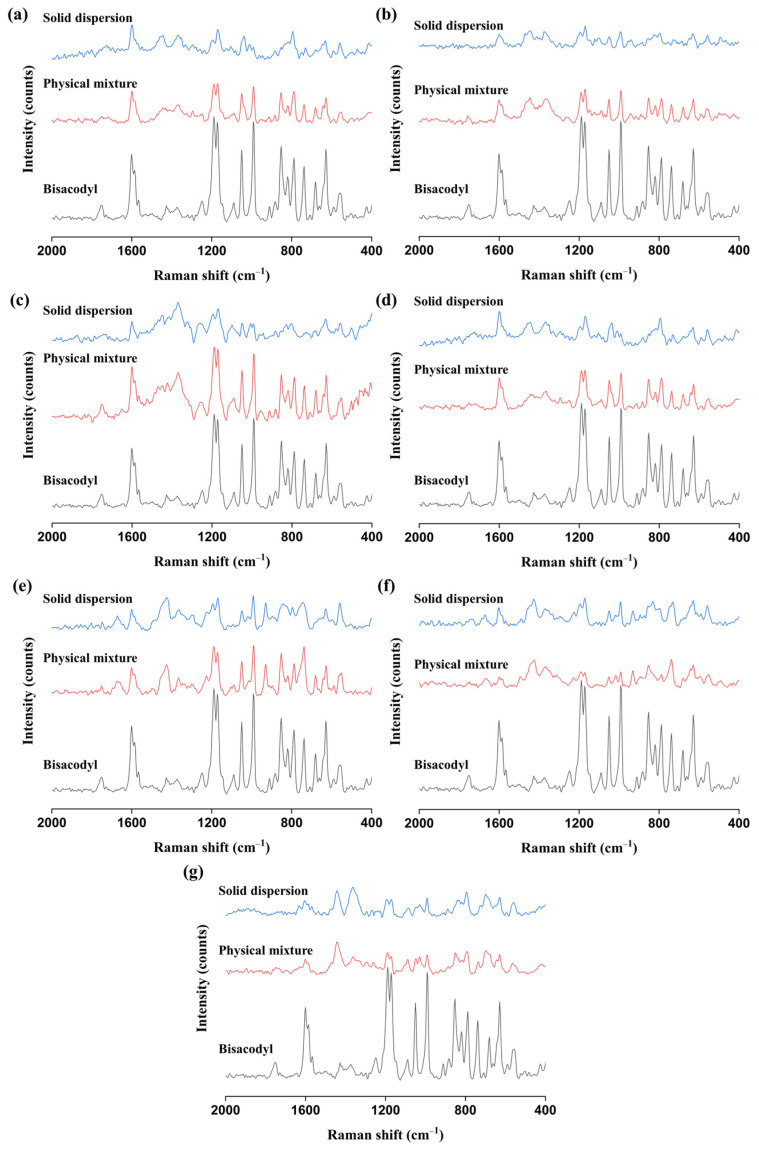
Raman spectra of pure bisacodyl, physical mixtures (drug:polymer = 1:2, *w*/*w*), and solid dispersions (drug:polymer = 1:2, *w*/*w*): (**a**) bisacodyl–HPC; (**b**) bisacodyl–HPMC; (**c**) bisacodyl–HPMCAS; (**d**) bisacodyl–HPMCP; (**e**) bisacodyl–PVP K12; (**f**) bisacodyl–PVP VA64; (**g**) bisacodyl–Soluplus^®^.

To confirm and identify the molecular interactions of the bisacodyl–polymer systems as observed in the FT-IR analysis, Raman spectroscopy was conducted to provide further complementary information on the molecular interactions [46]. The Raman spectra of bisacodyl, the physical mixtures, and the hot-melt-extruded solid dispersions are shown in Figure 5 and Table 4. The characteristic Raman peaks of bisacodyl were observed at 1601 cm^−1^ (C=O), 1189 cm^−1^ (C−O), and 1172 cm^−1^ (C−O), which are similar to reported values [47]. In all solid dispersions processed by hot-melt extrusion, Raman peak changes were observed; compared to those of pure bisacodyl and the physical mixture, the characteristic peak at 1189 cm^−1^ shifted to 1196 cm^−1^. This result is in good agreement with the FT-IR result, although the relative differences between the bisacodyl amorphous solid dispersion samples are not apparent. Additionally, the peak at 1172 cm^−1^ slightly shifted to 1169 cm^−1^ in the solid dispersions prepared with HPC, HPMC, HPMCAS, HPMCP, and PVP K12.

### 3.3. A Summary of the Assessment of Miscibility and Molecular Interaction

The assessment of miscibility and molecular interaction using various analytical methods and approaches is summarized in Table 5. The Hansen solubility parameter and single $T_{g}$ showed that bisacodyl and seven different polymers (HPC, HPMC, HPMCAS, HPMCP, PVP K12, PVP VA64, and Soluplus^®^) were miscible. Additionally, although differences were detected depending on the approaches and analysis methods, based on the comprehensive results of the various approaches, it was confirmed that there were molecular interactions between bisacodyl and all studied polymers. Therefore, because using only a single approach for assessing miscibility and molecular interactions could be very misleading, it is crucial that multiple approaches be applied to evaluate the miscibility and interaction between the drug and polymer [46].

### 3.4. Non-Sink Dissolution Test

**Figure 6:**
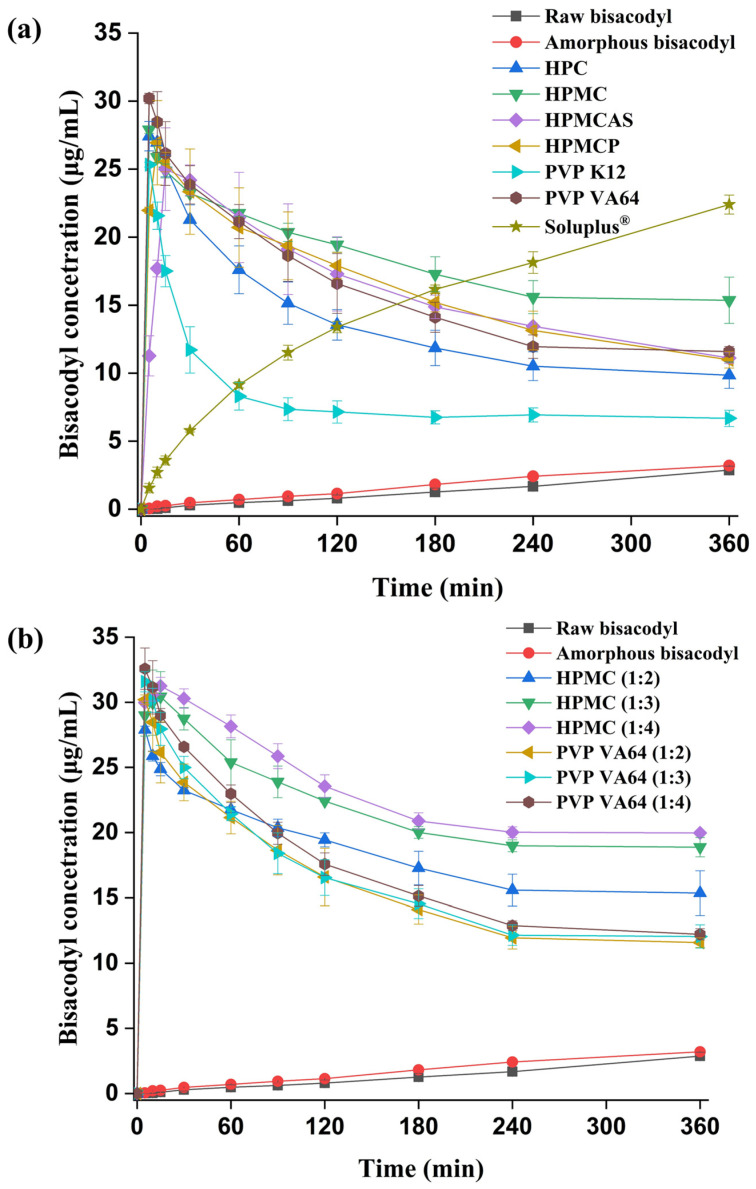
Effect of polymer type (**a**) and the ratio of HPMC and PVP VA64 (**b**) on the non-sink dissolution profiles of bisacodyl solid dispersions prepared by hot-melt extrusion (*n* = 3).

To assess and characterize the solubility capacity and performance of supersaturation, the in vitro dissolution tests of the hot-melt-extruded solid dispersions with seven polymers (HPC, HPMC, HPMCAS, HPMCP, PVP K12, PVP VA64, and Soluplus^®^) at a drug-to-polymer ratio of 1:2 were performed under non-sink conditions in pH 7.2 buffer, which was selected in consideration of the pH of the intestinal fluid. As shown in Figure 6a and Table S2, hot-melt-extruded amorphous solid dispersions prepared with the seven selected polymers, HPC, HPMC, HPMCAS, HPMCP, PVP K12, PVP VA64, and Soluplus^®^, at a 1:2 ratio showed a considerable increase in the concentration of dissolved bisacodyl compared to that of the crystalline bisacodyl. Overall, the spring effect of supersaturation was observed in all solid dispersions, which reached the maximum supersaturation concentration within 15 min, except for the Soluplus^®^-based solid dispersion. The spring effect is a faster initial dissolution of the drug, which increases the dissolution rate and extent of drug absorption [48]. The bisacodyl-containing solid dispersion with Soluplus^®^ showed a slow dissolution of bisacodyl without precipitation. This result for the Soluplus^®^-based solid dispersion might be related to delayed drug release, owing to the gel matrix formation of Soluplus^®^ in the dissolution medium [49,50]. The maximum concentrations of dissolved bisacodyl in solid dispersions with HPC, HPMC, HPMCAS, HPMCP, PVP K12, and PVP VA64 were obtained as 27.42 μg/mL, 27.90 μg/mL, 25.00 μg/mL, 26.94 μg/mL, 25.33 μg/mL, and 30.20 μg/mL, respectively. The maximum concentration of dissolved bisacodyl in the solid dispersion with PVP VA64 was approximately 10 times higher than that in the crystalline bisacodyl. Among the bisacodyl-containing solid dispersions, the maximum concentration of bisacodyl was the highest in the PVP VA64–based solid dispersion, followed by HPMC (*p <* 0.05). In particular, the time to reach the maximum supersaturation concentration was very fast, within 5 min, for the bisacodyl amorphous solid dispersion containing HPC, PVP VA64, or PVP K12, along with HPMC. In addition, in all solid dispersions, except for the formulation with Soluplus^®^, the concentration of the dissolved bisacodyl decreased as the supersaturated bisacodyl recrystallized after reaching the maximum concentration. The concentrations of dissolved bisacodyl at 6 h in solid dispersions with HPC, HPMC, HPMCAS, HPMCP, PVP K12, and PVP VA64 were obtained as 9.85 μg/mL, 15.36 μg/mL, 11.11 μg/mL, 10.98 μg/mL, 6.68 μg/mL, and 11.58 μg/mL, respectively. In particular, the concentration of the dissolved drug in the PVP K12–based solid dispersion rapidly decreased, indicating that the effectiveness of PVP K12 in maintaining supersaturation was low. The rate of desupersaturation following the recrystallization was lowest in the solid dispersion with HPMC. These results for the dissolution profile of the solid dispersions are related to the inhibition of crystallization by the polymer, which is referred to as the parachute effect [51,52,53]. The parachute effect refers to the prolonged supersaturation of drugs by inhibiting their recrystallization and precipitation [48]. Therefore, solid dispersions with HPMC and PVP VA64, which showed high supersaturation and a low rate of desupersaturation, were prepared at ratios of 1:3 and 1:4 and additionally evaluated using the non-sink dissolution test.

The dissolution profiles of bisacodyl-containing solid dispersions at various drug–polymer ratios prepared with HPMC and PVP VA64 are presented in Figure 6b and Table S3. The concentrations of bisacodyl at 6 h in solid dispersions prepared with HPMC and PVP VA64 were obtained as 18.88 μg/mL and 12.05 μg/mL at a 1:3 ratio, respectively, and were 19.97 μg/mL and 12.22 μg/mL at a 1:4 ratio, respectively. This result indicated that the PVP VA64–based solid dispersion showed an insignificant effect on precipitation inhibition as the drug:polymer ratio increased from 1:2 to 1:4 (*p* > 0.05). In contrast, the solid dispersion prepared with HPMC showed a significant improvement in precipitation inhibition as the ratio of HPMC increased from 1:2 to 1:4 (*p <* 0.05), which may be related to the steric hindrance effect causing the large negative deviation between the theoretical and experimental values of $T_{g}$. These results indicate that HPMC was a better precipitation inhibitor than PVP VA64. In addition, these results may be related to the absence of conversion to the crystalline form in the DVS-treated HPMC solid dispersion and the result of showing a characteristic peak in the DVS-treated PVP VA64 solid dispersion. HPMC is an effective polymer in maintaining supersaturation [54,55,56]. Therefore, based on the results of various approaches, it was confirmed that HPMC, which showed excellent solubility, miscibility, and interaction with bisacodyl, is a suitable polymer for preparing bisacodyl-containing solid dispersions processed by the hot-melt extrusion process. Afterwards, bisacodyl solid dispersion at a ratio of drug:HPMC = 1:4 was selected as the optimal solubilized sample for enteric-coated tablet production, in vitro tablet dissolution, and in vivo efficacy evaluation.

### 3.5. In Vitro Dissolution Test of Enteric-Coated Tablet Containing Bisacodyl Solid Dispersions

**Figure 7:**
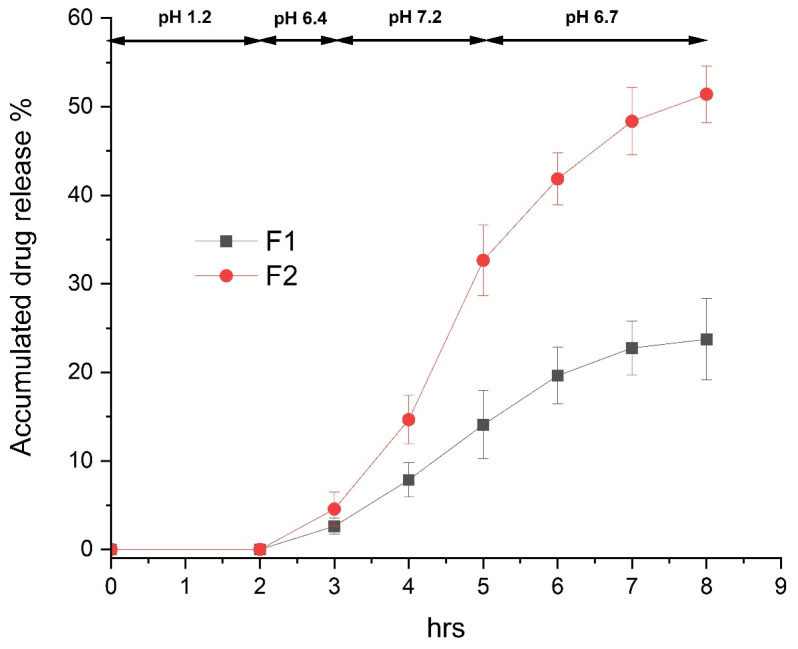
In vitro drug release profile of enteric-coated tablet containing raw bisacodyl (F1) and hot-melt-extruded bisacodyl solid dispersion (F2) in continuous dissolution medium, considering the general physiological condition of the gastrointestinal tract (*n* = 6, mean value ± SD).

The in vitro drug release profile evaluated in continuous dissolution medium is shown in Figure 7. No detectable drug release in pH 1.2 for 2 h was observed for the enteric-coated tablets of F1 and F2 containing raw bisacodyl and hot-melt-extruded bisacodyl solid dispersion, respectively. The drug release was initiated at pH 6.4, since the used enteric coating materials, Eudragit L and S (methacylic acid/ethyl acrylate copolymer) typically can start to dissolve at pH 5.5 and higher. However, the drug released percent was very low, below 5% for both F1 and F2 tablets, due to sufficient enteric-coating thickness for the inhibition of drug release. The accumulated drug release percent dramatically increased to over 30% and 50% for the F2 tablet at pH 7.2 and pH 6.7, respectively. This is because solubilized bisacodyl can be quickly released from the tablet and the dissolution of the coating material can be accelerated at a higher pH condition. In contrast, drug release from the F1 tablet was markedly lower than F2, releasing below 15% and 25% at pH 7.2 and pH 6.7, respectively, which is due to poor water solubility of the raw bisacodyl in the F1 tablet. This result suggests that solid bisacodyl that does not dissolve in the intestine fluid for F1 could be excreted with feces without therapeutic efficacy.

### 3.6. In Vivo Therapeutic Efficacy in Constipation-Induced Rabbits

**Figure 8:**
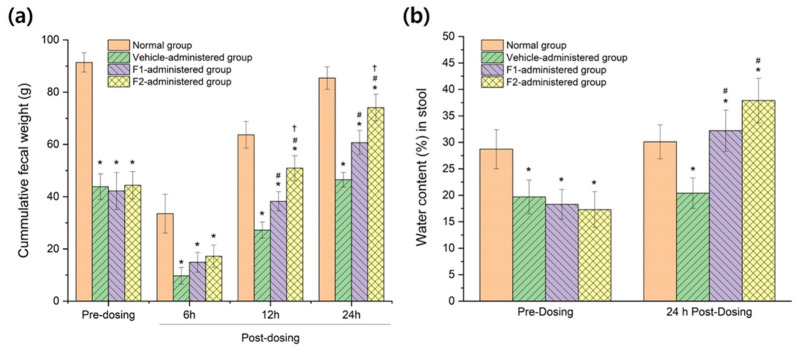
In vivo therapeutic efficacy evaluation results; the effect of bisacodyl formulation administration on (**a**) the fecal weight and (**b**) the water content of the stool in constipation-induced rabbits (*n* = 8, mean ± SD). The normal group consists of normal rabbits administered only water. The vehicle-administered group is a group administered enteric-coated tablets containing a drug-free vehicle. The F1-administered group is a group administered enteric-coated tablets containing raw bisacodyl. The F2-administered group is a group administered enteric-coated tablets containing a solubilized amorphous solid dispersion of bisacodyl (drug:HPMC=1:4). * indicates *p <* 0.05 versus normal group, observed through ANOVA (Analysis of Variance). # indicates *p <* 0.05 versus vehicle-administered group, observed through ANOVA. † indicates *p <* 0.05 versus the F1-aadministered group, observed through ANOVA.

Figure 8 shows a statistically significant reduction in fecal excretion (Figure 8a) and water content in stool (Figure 8b) for constipation-induced rabbits compared to normal rabbits (*p <* 0.05). In constipation-induced rabbits, no significant increase in fecal weight and water content in stool was observed for the group administered a vehicle that did not contain bisacodyl, compared to before administration. In contrast to the vehicle-administered group, groups F1 and F2, containing bisacodyl, began to display an increase in fecal weight at 6 h postdosing; then, both fecal weight and water content increased significantly, with a clear difference at 24 h after administration. In particular, it was shown that the therapeutic efficacy for F2, containing a solubilized bisacodyl solid dispersion, was significantly improved compared to F1, containing raw bisacodyl with poor water solubility. This is consistent with the expectation based on the in vitro dissolution results. This in vivo result suggests that the prepared enteric-coated tablet can be effectively delivered to the large intestine; the solubilized bisacodyl retained in the F2 tablet would be quickly liberated into the intestinal fluids after erosion of the enteric coating layer, even in the harsh condition of the large intestine, hence immediately inducing its pharmacological laxative action.

## 4. Conclusions

In this study, various analytical approaches were used as pre-formulation strategies to evaluate the miscibility and interaction between drug and polymer to select a suitable polymer for manufacturing amorphous solid dispersions processed by the hot-melt extrusion process. Seven different polymers (HPC, HPMC, HPMCAS, HPMCP, PVP K12, PVP VA64, and Soluplus^®^), which were selected based on their thermal properties, such as $T_{g}$ and degradation temperature, were used to evaluate the miscibility and interaction with bisacodyl. The Hansen solubility parameter was calculated, and single $T_{g}$ was identified using MDSC analysis to investigate the miscibility between bisacodyl and the seven polymers. The results of the miscibility test showed that all selected polymers exhibited miscibility with bisacodyl. In addition, DVS, FT-IR, Raman spectroscopy, and a comparison of the predicted and experimental $T_{g}$ values were conducted to assess the molecular interaction between bisacodyl and the polymers. Based on the comprehensive results of DVS, FT-IR, and Raman analyses, all investigated polymers showed molecular interactions with bisacodyl. Furthermore, from the results of PXRD analysis for DVS-treated solid dispersions, the stability of solid dispersions with HPMC, HPMCAS, HPMCP, and Soluplus^®^ was ensured. In particular, among the seven different polymers, HPMC showed a relatively large deviation between the predicted and experimental values of $T_{g}$, which may be attributed to strong steric hindrance. These results would be related to the results of the non-sink dissolution test for the HPMC-based solid dispersion, which showed an excellent inhibitory effect on recrystallization, depending on the ratio of the polymer. Furthermore, the in vitro dissolution test and in vivo animal study showed that the solubilized bisacodyl solid dispersion via hot-melt extrusion can be released very quickly into the intestinal fluids from the tablet, hence immediately inducing its enhanced pharmacological laxative action. Therefore, it can be concluded that the various analytical approaches described in this study are successfully proposed as useful pre-formulation strategies for the improvement of therapeutic efficacy using the solubilization of poorly soluble drugs through selecting the optimal polymer for manufacturing amorphous solid dispersions processed by the hot-melt extrusion process.

## Figures and Tables

**Table 1:** Composition of the enteric-coated tablet formulation.

Content	F1 (mg/Tablet)	F2 (mg/Tablet)
Tablet		
Raw bisacodyl	2.5	
Hot-melt-extruded solid dispersions (Bisacodyl:HPMC = 1:4 *w*/*w*)		12.5
Aerosil 200	10	10
Avicel PH102	40	30
HPC-EXF	5	5
Kollidon CL	5	5
Magnesium stearate	1.25	1.25
Enteric-coating layer		
Eudragit L100	13.75	13.75
Eudragit S100	13.75	13.75
TEC	2.25	2.25
Talc	6.5	6.5
Total weight of enteric-coated tablet	100	100

**Table 2:** The calculated solubility parameters and interaction parameters using Hansen group contribution theory for bisacodyl and the polymers.

Compound/Polymer	Solubility Parameter (MPa^1/2^)	∆δ (MPa^1/2^)	Interaction Parameter
Bisacodyl	26.2		
HPC	22.4	3.8	1.29
HPMC	23.7	2.5	0.56
HPMCAS	29.1	2.9	0.75
HPMCP	22.4	3.8	1.29
PVP K12	19.4	6.8	4.13
PVP VA64	21.1	5.1	2.32
Soluplus^®^	23.4	2.8	0.70

**Table 3:** Peak positions of different groups in the FT-IR spectra for bisacodyl and its solid dispersions (drug:polymer = 1:2, *w*/*w*).

Bisacodyl (cm^−1^)	Bisacodyl–HPC (cm^−1^)	Bisacodyl–HPMC (cm^−1^)	Bisacodyl–HPMCAS (cm^−1^)	Bisacodyl–HPMCP (cm^−1^)	Bisacodyl–PVP K12 (cm^−1^)	Bisacodyl–PVP VA64 (cm^−1^)	Bisacodyl–Soluplus^®^ (cm^−1^)	Assignment
1757	1760	1759	1754	1759	1757	1758	1759	C=O group
1506	1506	1507	1506	1507	1506	1506	1506	Aromatic group
1465	-	-	-	-	1465	1464	1461	Aromatic group
1431	-	-	-	-	1432	1435	1434	Aromatic group
1218	-	-	-	-	1218	-	-	C−O group
1208	-	-	-	-	1207	-	-	C−O group

**Table 4:** Peak positions of different groups in the Raman spectra for bisacodyl and its solid dispersions (drug:polymer = 1:2, *w*/*w*).

Bisacodyl (cm^−1^)	Bisacodyl–HPC (cm^−1^)	Bisacodyl–HPMC (cm^−1^)	Bisacodyl–HPMCAS (cm^−1^)	Bisacodyl–HPMCP (cm^−1^)	Bisacodyl–PVP K12 (cm^−1^)	Bisacodyl–PVP VA64 (cm^−1^)	Bisacodyl– Soluplus^®^ (cm^−1^)	Assignment
1601	1601	1601	1601	1601	1601	1601	1603	C=O group
1189	1196	1196	1196	1196	1196	1196	1196	C−O group
1172	1169	1169	1169	1169	1169	1172	1172	C−O group

**Table 5:** Summary of evaluation of miscibility, interaction, and stability between bisacodyl and the polymers using various approaches.

Systems	Miscibility		Molecular Interaction				Stability
	Solubility Parameter	$\mathrm{Sin}\mathrm{gle}\;T_{g}$	$T_{g}$ Deviation	DVS	FT-IR	Raman	DVS and PXRD
Bisacodyl–HPC	Miscible	-	-	No	Yes	Yes	Not stable
Bisacodyl–HPMC	Miscible	Miscible	Negative	Yes	Yes	Yes	Stable
Bisacodyl–HPMCAS	Miscible	Miscible	Negative	Yes	Yes	Yes	Stable
Bisacodyl–HPMCP	Miscible	Miscible	Negative	Yes	Yes	Yes	Stable
Bisacodyl–PVP K12	Miscible	Miscible	Negative	Yes	No	Yes	Not stable
Bisacodyl–PVP VA64	Miscible	Miscible	Negative	Yes	Yes	Yes	Not stable
Bisacodyl–Soluplus^®^	Miscible	Miscible	Negative	Yes	Yes	Yes	Stable

## Data Availability

The data presented in this study are available in the article or supplementary materials.

## Supplementary Materials

The following supporting information can be downloaded at: https://www.mdpi.com/article/10.3390/pharmaceutics15122704/s1, Table S1: Generally used polymers in hot-melt extrusion for preparing solid dispersion; Table S2: Details of the calculation of Hansen solubility parameter of bisacodyl; Table S3: Non-sink dissolution data (concentration, μg/mL) of raw bisacodyl and bisacodyl-containing solid dispersion in pH 7.2 buffer; Table S4: Non-sink dissolution data (concentration, μg/mL) of bisacodyl-containing solid dispersion with HPMC and PVP VA64 in pH 7.2 buffer; Figure S1: DSC thermogram of amorphous bisacodyl; Figure S2: PXRD patterns of bisacodyl and solid dispersion prepared by hot-melt extrusion; Figure S3: Water sorption isotherms of amorphous bisacodyl and polymers. References [15,57] are cited in the supplementary materials.
