# Supplementary material for: Preparation of Hot-Melt-Extruded Solid Dispersion Based on Pre-Formulation Strategies and Its Enhanced Therapeutic Efficacy
Source: Pharmaceutics. 2023 Nov 30;15(12):2704. doi: 10.3390/pharmaceutics15122704 (PMC10747747; doi:10.3390/pharmaceutics15122704)
Supplement: Supplementary file 1 [file pharmaceutics-15-02704-s001.zip › pharmaceutics-2714601-supplementary.pdf]

Supplementary material

Preparation of Hot-Melt-Extruded Solid Dispersion Based on Pre-Formulation Strategies and Its Enhanced Therapeutic Efficacy

Seong-Kwang Lee [1,†], Eun-Sol Ha [1,†], Heejun Park [2], Kyu-Tae Kang [2], Ji-Su Jeong [1], Jeong-Soo Kim [3], In-hwan Baek [4] and Min-Soo Kim [1,*]

[1] College of Pharmacy, Pusan National University, 63 Busandaehak-ro, Geumjeong-gu, Busan 46241, Republic of Korea
[2] College of Pharmacy, Duksung Women's University, 33, Samyangro 144-gil, Dobong-gu, Seoul 01369, Republic of Korea
[3] Dong-A ST Co., Ltd., Giheung-gu, Yongin 17073, Republic of Korea
[4] College of Pharmacy, Kyungsung University, 309, Suyeong-ro, Nam-gu, Busan 48434, Republic of Korea; baek@ks.ac.kr
* Correspondence: minsookim@pusan.ac.kr; Tel.: +82-51-510-2813
† These authors contributed equally to this work.

Table S1. Generally used polymers in hot-melt extrusion for preparing solid dispersion.

**Polymer**	**Trade name**	**T_g (°C)**	**T_{deg} (°C)**	**Water soluble?**
Copolymer of poly(butyl methacylate), poly((2-dimethylaminoethyl) methacrylate) and poly(methyl methacrylate) 1:2:1	Eudragit® E PO	55	> 250	Yes at pH < 5
Copolymer of poly(methyl acrylate) and poly(methyl methacrylate) 1:1	Eudragit® L	111	> 170	Yes at pH > 7
Copolymer of poly(methyl acrylate) and poly(methyl methacrylate) 1:2	Eudragit® S	173	173	Yes at pH > 7
Copolymer of poly(ethyl acrylate) poly(methyl methacrylate) and poly(trimethylammonioethyl methacrylate chloride) 1:2:0.2	Eudragit® RL	65	170	No
Copolymer of poly(ethyl acrylate), poly(methyl methacrylate) and poly(trimethylammonioethyl methacrylate chloride) 1:2:0.1	Eudragit® RS	65	170	No
Methyl cellulose	Methocel®	190	250	Yes
Ethyl cellulose	Ethocel®	130	300	No
Hydroxypropyl cellulose	HPC SSL	81.8	> 220	Yes
Hydroxypropyl methylcellulose	Pharmacoat® 603	136.4	> 300	Yes
Hydroxypropyl methylcellulose acetate succinate	AQOAT® AS	121.6	250	Yes at pH >5.5
Hydroxypropyl methylcellulose phthalate	HP-50	135	190	Yes at pH >5
Poly(vinyl pyrrolidone)	Kollidon® 12	100	290	Yes
Poly(vinyl pyrrolidone)	Kollidon® 17	142	> 210	Yes
Copolymer of poly(methyl acrylate) and poly(ethyl acrylate) 1:1	Kollicoat® MAE100P	114	175	Yes at pH >5.5
Poly(ethylene glycol) grafted with poly(vinyl alcohol)	Kollicoat® IR	45	200	Yes
Poly(ethylene glycol) grafted with poly(vinyl alcohol) + Poly(vinyl alcohol)	Kollicoat® Protect	45	200	Yes
Copolymer of poly(vinyl pyrrolidone) and poly(vinyl acetate	Kollidon® VA64	105	> 220	Yes
Poly(ethylene glycol) grafted with a copolymer of poly(vinyl caprolactam) and poly(vinyl acetate)	Soluplus®	70	> 250	Yes

Table S2. Details of the calculation of Hansen solubility parameter of bisacodyl.

Group	Group number	F_{di} (MJ/m^3)$^{0.5}$/mol	F_{pi}^2 ((MJ/m^3)$^{0.5}$/mol)^2	E_{hi} (J/mol)
CH_3	2	840	0	0
CH	1	80	0	0
-CH=	4	800	0	0
>C=	1	70	0	0
Phenylene (o)	2	2540	24200	0
COO	2	780	480200	14000
-N=	1	20	640000	5000
Ring	1	190	0	0
Total		5320	1144400	19000
			Solubility parameter	
			δ_d (MPa$^{0.5}$)	24.05
			δ_p (MPa$^{0.5}$)	4.84
			δ_h (MPa$^{0.5}$)	9.27
			δ (MPa$^{0.5}$)	26.22

Molar volume was taken from the literature [S2].

Table S3. Non-sink dissolution data (concentration, μg/mL) of raw bisacodyl and bisacodyl-containing solid dispersion in pH 7.2 buffer.

Solid dispersions	0.083 h	0.167 h	0.25 h	0.5 h	1 h	1.5 h	2 h	3 h	4 h	6 h
Raw bisacodyl	0.02 ± 0.01[a]	0.03 ± 0.01	0.10 ± 0.02	0.29 ± 0.10	0.48 ± 0.20	0.63 ± 0.20	0.81 ± 0.23	1.27 ± 0.28	1.68 ± 0.25	2.88 ± 0.29
Amorphous bisacodyl	0.05 ± 0.01	0.21 ± 0.05	0.25 ± 0.03	0.47 ± 0.08	0.71 ± 0.12	0.95 ± 0.10	1.15± 0.15	1.82 ± 0.19	2.42 ± 0.21	3.21 ± 0.23
HPC	27.42 ± 1.08	26.95 ± 0.22	25.23 ± 0.79	21.29 ± 1.08	17.60 ± 1.76	15.15 ± 1.56	13.55 ± 1.12	11.85 ± 1.30	10.51 ± 1.06	9.85 ± 0.97
HPMC	27.90 ± 0.26	25.87 ± 0.38	24.87 ± 0.51	23.24 ± 0.21	21.76 ± 0.23	20.36 ± 0.67	19.44 ± 0.54	17.28 ± 1.29	15.59 ± 1.22	15.36 ± 1.71
HPMCAS	11.27 ± 1.48	17.70 ± 0.60	25.00 ± 3.04	24.19 ± 1.13	21.45 ± 3.31	19.13 ± 3.33	17.30 ± 2.73	14.88 ± 0.87	13.44 ± 0.13	11.11 ± 0.40
HPMCP	21.97 ± 0.23	26.94 ± 3.10	25.52 ± 0.71	23.35 ± 3.13	20.71 ± 2.92	19.38 ± 2.48	17.92 ± 0.96	15.21 ± 0.69	13.14 ± 1.43	10.98 ± 0.60
PVP K12	25.33 ± 0.18	21.58 ± 1.00	17.51 ± 1.15	11.71 ± 1.70	8.30 ± 1.01	7.35 ± 0.84	7.15 ± 0.82	6.75 ± 0.48	6.93 ± 0.52	6.68 ± 0.60
PVP VA64	30.20 ± 0.37	28.46 ± 2.24	26.15 ± 2.34	23.85 ± 1.41	21.15 ± 1.25	18.65 ± 1.89	16.60 ± 2.20	14.11 ± 1.11	12.87 ± 0.86	11.58 ± 0.38
Soluplus®	1.55 ± 0.34	2.70 ± 0.40	3.59 ± 0.33	5.78 ± 0.10	9.14 ± 0.23	11.51 ± 0.55	13.38 ± 0.41	16.17 ± 0.32	18.14 ± 0.80	22.40 ± 0.70

[a] Mean ± standard deviation (*n* = 3).

Table S4. Non-sink dissolution data (concentration, μg/mL) of bisacodyl-containing solid dispersion with HPMC and PVP VA64 in pH 7.2 buffer.

Solid dispersions (bisacodyl:polymer ratio)	0.083 h	0.167 h	0.25 h	0.5 h	1 h	1.5 h	2 h	3 h	4 h	6 h
HPMC (1:3)	28.99 ± 1.58[a]	29.98 ± 2.50	30.43 ± 1.93	28.74 ± 0.86	25.39 ± 1.73	23.89 ± 1.22	22.41 ± 0.06	20.01 ± 0.45	18.99 ± 0.45	18.88 ± 0.74
HPMC (1:4)	29.98 ± 0.11	30.58 ± 0.48	31.25 ± 0.67	30.29 ± 0.74	28.15 ± 0.87	25.86 ± 0.96	23.55 ± 0.88	20.89 ± 0.63	20.02 ± 0.41	19.97 ± 0.18
PVP VA64 (1:3)	31.58 ± 0.78	30.15 ± 0.93	27.95 ± 1.26	24.99 ± 0.86	21.44 ± 0.52	18.41 ± 1.56	16.55 ± 1.37	14.55 ± 1.14	12.15 ± 0.79	12.05 ± 0.89
PVP VA64 (1:4)	32.58 ± 1.59	31.15 ± 2.05	28.99 ± 0.54	26.58 ± 0.03	22.98 ± 0.67	19.95 ± 0.85	17.58 ± 0.87	15.15 ± 0.79	12.88 ± 0.37	12.22 ± 0.43

[a] Mean ± standard deviation (*n* = 3).

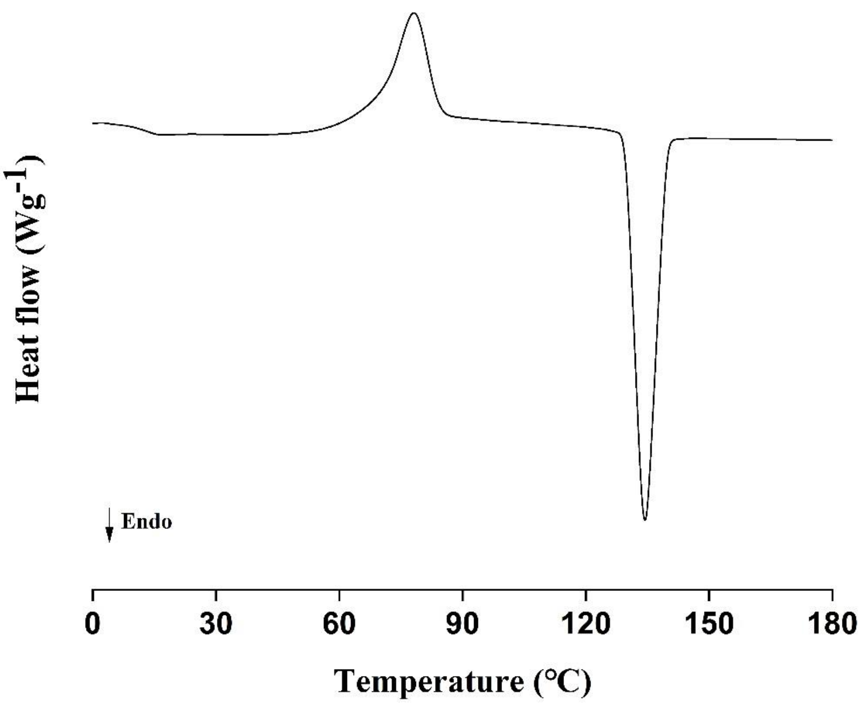

Figure S1. DSC thermogram of amorphous bisacodyl.

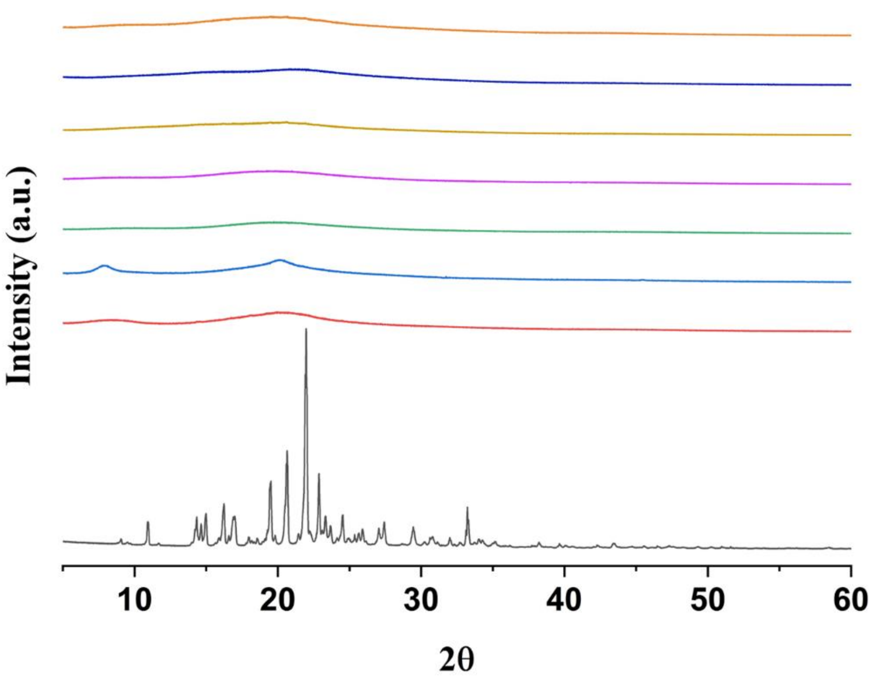

Figure S2. PXRD patterns of bisacodyl and solid dispersion prepared by hot-melt extrusion.

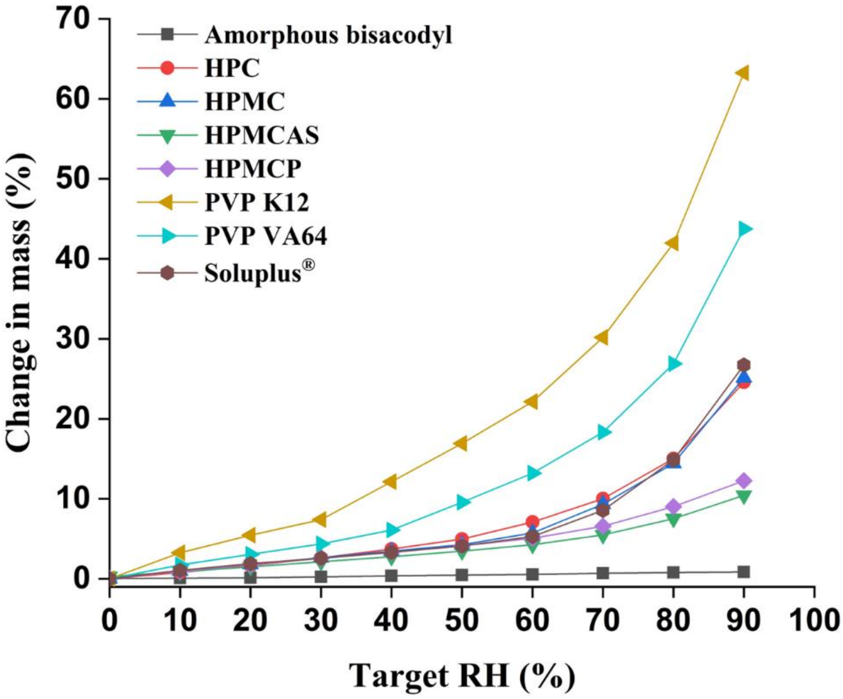

Figure S3. Water sorption isotherms of amorphous bisacodyl and polymers.

References

[S1]. Hansen, C.M. *Hansen Solubility Parameters. A User's Handbook*, 2nd ed.; CRC Press: Boca Raton, FL, USA, 2007; ISBN 0849372488.

[S2]. Lee, S.K.; Sim, W.Y.; Ha, E.S.; Park, H.; Kim, J.S.; Jeong, J.S.; Kim, M.S. Solubility of bisacodyl in fourteen mono solvents and N-methyl-2-pyrrolidone + water mixed solvents at different temperatures, and its application for nanosuspension formation using liquid antisolvent precipitation. J. Mol. Liq. 2020, 310, 113264.
